# Recent Chronology of COVID-19 Pandemic

**DOI:** 10.3389/fpubh.2022.778037

**Published:** 2022-05-04

**Authors:** Sazada Siddiqui, Heba Waheeb Saeed Alhamdi, Huda Ahmed Alghamdi

**Affiliations:** Department of Biology, College of Science, King Khalid University, Abha, Saudi Arabia

**Keywords:** SARS-CoV-2, COVID-19, SARS-CoV, MERS-CoV, pandemic

## Abstract

COVID-19 is highly contagious and is caused by severe acute respiratory syndrome coronavirus 2. It spreads by means of respiratory droplets and close contact with infected persons. With the progression of disease, numerous complications develop, particularly among persons with chronic illnesses. Pathological investigations indicate that it affects multiple organs and can induce acute respiratory distress syndrome. Prevention is vital and self-isolation is the best means of containing this virus. Good community health practices like maintaining sufficient distance from other people, wearing protective face masks and regular hand washing should be adopted. Convalescent plasma transfusion and the administration of the antiviral Remdesivir have been found to be effective. Vaccines offer lifesaving protecting against COVID-19 which has killed millions and our best bet for staying safe. Screening, suppression/containment as well as mitigation are the strategies implemented for controlling COVID-19 pandemic. Vaccination is essential to end the COVID-19 pandemic and everyone should have an access to them. The current COVID-19 pandemic brought the global economy to a standstill and has exacted an enormous human and financial toll.

## Introduction: Origins and Timeline

In December 2019, cases of an undetermined pneumonia were found in adults within Wuhan city, in the Hubei region of China; its clinical features closely resembled viral pneumonia. This pneumonia was linked to a Huanan seafood marketplace in Wuhan where various kinds of livestock (e.g., bats, snakes, groundhogs, and poultry) are traded. A surveillance mechanism installed during the severe acute respiratory syndrome (SARS) outbreak was restarted, and respiratory samples of infected persons were sent to laboratories for etiological examination. A considerable number of tests were undertaken to detect the cause of this disease; in the process, numerous etiological agents that would suggest analogous indications like avian influenza virus, Middle East respiratory syndrome coronavirus (MERS-CoV), SARS coronavirus (SARS-CoV), and various other common respiratory diseases were ruled out. Upon analyzing the results of those respiratory tests, specialists at the PRC Centers for Disease Control stated that the cause of this outbreak was a novel coronavirus with more than 70% homology with SARS-CoV and more than 95% similarity with bat coronavirus (1). On December 31, 2019, China informed the World Health Organization (WHO) of this outbreak, and on January 1, 2020, the Huanan seafood marketplace was closed. Samples drawn from this marketplace were found to be positive. There was an exponential increase in the number of reported cases, several of whom had not even gone to that marketplace; this suggested human-to-human transmission (1). On January 11, 2020, the first fatal case was registered. On January 12, 2020, WHO named this novel virus COVID-19; the International Committee on Taxonomy of Viruses named this virus severe acute respiratory syndrome coronavirus 2 (SARS-CoV-2). A SARS expert, Professor Zhong Nanshan, reported that on his visit to Wuhan on January 20, 2020, COVID-19 was proliferating within the populace; on that same day, it was reported that health care professionals who had been caring for patients had become infected (2). A general closure was declared in Wuhan on January 23, 2020 to inhibit virus proliferation.

Wuhan with a population of around 11 million is a key transportation hub, and had seen a large press of travelers between late December 2019 and February 2020, on account of the Spring Festival (3). While the government of China made considerable efforts to regulate the movement of people, the virus moved swiftly from Wuhan to other Chinese cities, and also to other countries, probably via asymptomatic carriers (4). The WHO declared the outbreak a public health emergency of global concern on January 30, 2020. Table 1 mentions the timeline of COVID-19 (5). As of time of writing, the pandemic is ongoing and has continued to impose a considerable challenge worldwide. So global is this threat that all countries must show solidarity and cooperation to contain its spread.

**Table 1:** Timelines of COVID-19.

**Date**	**Events**
1 December 2019	In Wuhan healthcare center, in China, first COVID-19 patient was detected.
31 December 2019	People in Wuhan were alerted about the warning signs of a pneumonia epidemic by the Wuhan authorities.
7 January 2020	A newly discovered virus known as coronavirus has been confirmed by Chinese researchers.
10 January 2020	WHO has made a comprehensive collection of online clinical guidance for each nation about how to detect, monitor, and cure suspected patients. For an emergency, such guidance was also reviewed with WHO Provincial Officers. Duties and assignments to be conveyed to WHO members in countries.
11 January 2020	First coronavirus-related death was reported in Wuhan.
13 January 2020	First COVID-19 case outside China was reported in Thailand.
21 January 2020	First active case of COVID-19 was identified in United States.
23 January 2020	Wuhan was shut down by Chinese authorities.
24 January 2020	First COVID-19 case was declared in France and United Kingdom.
30 January 2020	WHO has declared the virus as an international health crisis, with over 9,000 patients reported globally. India declared its first COVID-19 case.
1 February 2020	First COVID-19 case was identified in Spain.
2 February 2020	Coronavirus was found to be the cause of the first fatality outside China.
28 February 2020	First COVID-19 case was identified in New Zealand.
29 February 2020	Coronavirus was found to be the cause of the first death in the United States.
11 March 2020	WHO declared the disease outbreak caused by novel coronavirus as pandemic.
19 March 2020	There have been no newly identified cases in China since pandemic began.
24 March 2020	Olympics was postponed till 2021 due to outbreak.
1 April 2020	A six-week-old baby died of coronavirus.
2 April 2020	COVID-19 cases cross one million as per John Hopkins report.
8 April 2020	Wuhan reopened after a lockdown of 76 days.
11 April 2020	Global COVID-19 cases have reached 1.7 million with 100,000 fatalities. In the United States, COVID-19 deaths have reached 20,000, highest in world.
20 April 2020	Global market crashed, causing oil prices in the United States to fall in negative.
28 April 2020	COVID-19 cases in United States crossed 1 million.
7 May 2020	WHO cautions that if COVID-19 is not contained, it can kill around 83,000 to 190,000 humans in African countries by 2020.
27 May 2020	Greater than 100,000 fatalities reported across United States.
2 June 2020	For first time, Wuhan did not record any new case.
8 June 2020	New Zealand has declared itself a COVID-19-free nation.
11 June 2020	COVID-19 cases in United States crossed 2 million.
3 July 2020	Global COVID-19 cases touched 11 million.
7 July 2020	United States formally withdraws from WHO. President of Brazil got infected with COVID-19
21 July 2020	European leaders have agreed to establish a €750 billion recovery fund for rebuilding European union economies ruined by COVID-19.
11 August 2020	Russian President reveals that Sputnik-V vaccine has been licensed for public usage in Russia, even though Phase 3 trials, which are generally required before clearance, have yet to be completed. Sputnik-V vaccine is being developed by Gamaleya Institute in Moscow.
15August 2020	Production of Sputnik-V began in Russia.
23August 2020	FDA grants an emergency use approval for using convalescent plasma to treat COVID-19.
27 August 2020	The Centers for Disease Control and Prevention (CDC) has informed public health officials across the United States to make preparation for distribution of coronavirus vaccine by late Oct. 2020.
4 September 2020	The Lancet has published the first peer-reviewed outcomes of Phase 1 and Phase 2 clinical trials of Sputnik-V vaccine.
2 October 2020	Trump and first lady Melania Trump have tested +ive for COVID-19.
12 October 2020	Advance clinical trials of Johnson & Johnson's coronavirus vaccine have been halted due to an unexplainable sickness of a volunteer.
10 December 2020	The FDA's vaccine advisors voted to advise agency to grant emergency use approval to Pfizer and BioNTech's vaccine.
18 December2020	Moderna's coronavirus vaccine has been approved for emergency use by FDA.
14 January 2021	WHO team entrusted with determining the cause of the disease outbreak in Wuhan reached China.
20 January 2021	U S withdrawal from WHO has been halted by US President Joe Biden.
22 February 2021	In US, fatalities from COVID-19 have surpassed 500,000.
27 February 2021	Johnson & Johnson's COVID-19 vaccine, the first single dose COVID-19 vaccine accessible in the United States, has been granted emergency use approval by FDA.
30 March 2021	As per a 120-page WHO report, the novel coronavirus which causes COVID-19 likely to spread to human beings via an animal and likely began circulating in human beings not more than a month or two before it was discovered in Dec. 2019.
17 April 2021	As per Johns Hopkins report, the global death toll from COVID-19 has surpassed 3 million.
3 August 2021	Highly infectious Delta variant is responsible for an approximate 93.4 percent of coronavirus spreading in the United States during the last 2 weeks in July, as per CDC estimates.
12 August 2021	For a few immunocompromised persons, FDA has approved a booster COVID-19 vaccination dose.
23 August 2021	Pfizer/BioNTech vaccine is the first coronavirus vaccine to receive FDA approval for persons aged 16 and above.
7 October 2021	Pfizer and BioNTech have filed an emergency use approval of their COVID-19 vaccine with FDA for children aged 5 to 11.
1 November 2021	Global death toll from COVID-19 surpasses 5 million.
2 November 2021	Director of US CDC Dr. Rochelle Wilensky, endorses a recommendation for vaccinating children against COVID-19, aged 5–11.
3 November 2021	WHO issues emergency use approval for Bharat Biotech's Covaxin.
4 November 2021	Novavax completes procedure for emergency use authorization of its COVID-19 vaccine with WHO.
19 November 2021	Booster shots of Pfizer/BioNTech and Moderna COVID-19 vaccines for all adults, authorized by FDA, also endorsed by CDC.
24 November 2021	From South Africa, B.1.1.529 variant was first reported to WHO.
26 November 2021	WHO stated the variant B.1.1.529 as a variant of concern, called it Omicron. Europe's first case of new Omicron variant confirmed in Belgium.
27 November 2021	UK, Italy, and Germany confirmed their 2 cases of new Omicron variant.
28 November2021	Denmark reported 2 cases of new Omicron variant in travellers from South Africa.

## Etiology

**Figure 1:**
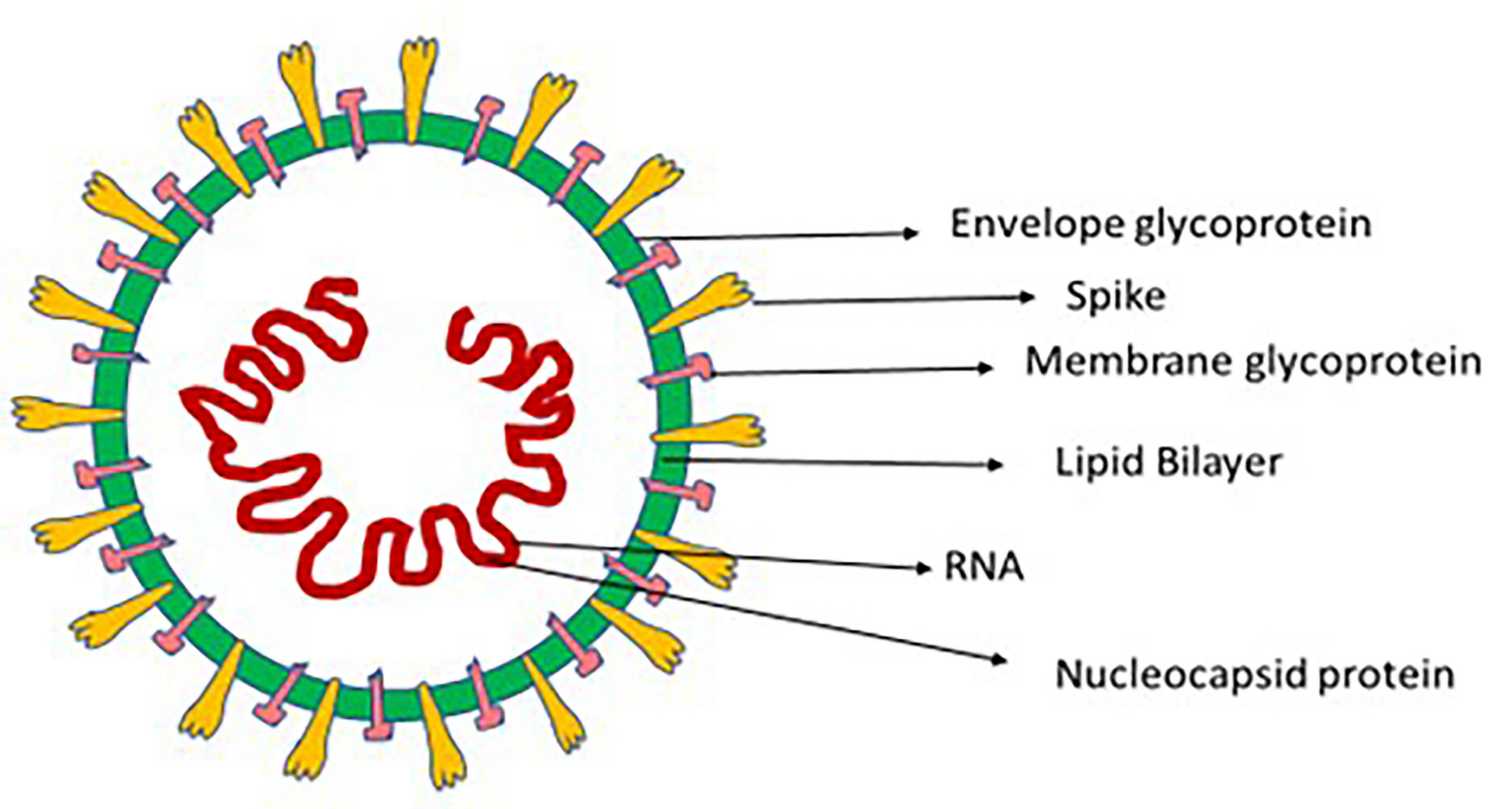
Composition of coronavirus instigating respiratory disease in human beings.

Coronaviruses (CoV) comprise a large family of single positive-stranded RNA viruses that, when seen under an electron microscope, resemble a crown; this appearance comes from its envelope, which features spike-like glycoproteins. When Tyrell and Bynoe reported in 1966 on coronaviruses (after isolating them from people infected by the common cold), they called them “coronaviruses” on account of the presence of spherical virions with a core shell and surface projections reminiscent of a solar corona (6). The Latin word for crown is *coronam* (Figure 1).

Coronaviruses belong to the Nidovirales order, which comprises the Roniviridae, Arteriviridae, and Coronaviridae families; the Coronaviridae family, in turn, is divided into the Torovirinae and Coronavirinae subfamilies. The Coronavirinae subfamily is genetically subclassified into the alpha CoV (*Alphacoronavirus*), beta CoV (*Betacoronavirus*), gamma CoV (*Gammacoronavirus*), and delta CoV (*Deltacoronavirus*) genuses (7). Moreover, the *Betacoronavirus* genus is further divided into five lineages. These subtypes are categorized on the basis of phylogenetic clustering, and their RNA genome varies in length from 26 to 32 kilobases (8). As per genomic examinations, rodents and bats are the genetic sources of *Alphacoronavirus* and *Betacoronavirus*, and avian species of *Gammacoronavirus* and *Deltacoronavirus* (9). Respiratory diseases in human beings and gastroenteritis illness in animals are generally caused by *Alphacoronavirus* and *Betacoronavirus*; diseases in avian species, on the other hand, are mostly caused by *Gammacoronavirus* and *Deltacoronavirus* types, although a few of them can also cause infections in mammals (10). Six kinds of coronavirus have been detected in human beings (HCoVs): HCoV-229E and HCoV-NL63 are *Alphacoronavirus* types, while HCoV-HKU1, HCoV-OC43, SARS-CoV, and MERS-CoV are *Betacoronavirus* types (11). Coronaviruses are important pathogens on account of their high infection rates and their extent of proliferation. Approximately 5–10% of severe respiratory infections are caused by coronaviruses, and around 2% of the world's population comprises carriers. The coronaviruses generally found in human beings are *Betacoronavirus* types HCoV-HKU1 and HCoV-OC43, and *Alphacoronavirus* types HCoV-NL63 and HCoV-229E. Among immunocompetent persons, besides causing the common cold, these viruses can cause minor upper-respiratory illnesses; SARS-CoV-2, SARS-CoV, and MERS-CoV, on the other hand, cause acute respiratory illnesses (12). SARS-CoV-2, the virus responsible for the COVID-19 pandemic, is a *Betacoronavirus*. Its gene characterization shows an 89% and 82% nucleotide homology with SARS-like bat coronaviruses and SARS-CoV respectively (13, 14). On the basis of these outcomes, the novel coronavirus has been called SARS-CoV-2. This virus's RNA genome length ranges from 29,891 to 29,903 nucleotides, and it is sensitive to heat and light (9). Moreover, it can be successfully deactivated by contact with lipid solvents like peroxyacetic acid, ether (75%), ethanol (60%), and disinfectants that contain chlorine.

## SARS-CoV-2 Variants of Concern (VOCs) and Variants of Interest (VOIs)

SARS-CoV-2 is susceptible to genetic evolution, ensuing numerous variants having distinct attributes from the original strain. In a pandemic, frequent genomic sequencing of samples is vital, because it aids in the detection of new SARS-CoV-2 variants. Initially genetic evolution was least with the worldwide prevalence of D614G variant having higher transmission rate but lacking the capability to induce acute illness (15). In human beings, another variant in Denmark, was detected which is linked to farmed minks which got infected, although it was not linked to higher transmissibility (16). After that, numerous variants have been detected, with a few being classified as variants of concern (VOCs) because of increased virulence or transmissibility, decreased neutralization of antibodies gained from vaccination or infection, the capability to avoid detection or a reduction in therapeutic or efficacy of vaccines. With the continual emergence of numerous variants, WHO and CDC (Centers for disease control and prevention) have developed their own categorization system for differentiating the new evolving variants into variants of interest (VOIs) and variants of concern (VOCs).

## Variants of Concern (VOCs)

### Alpha (B.1.1.7)

B.1.1.7 lineage, commonly known as Alpha variant, was discovered in United Kingdom in late Dec. 2020, on the basis of sequencing of whole-genome samples from COVID-19 patients (17, 18). Alpha variant started spreading in United Kingdom in Sep. 2020 and it was stated to be 43 percent to 82 percent more contagious than previous SARS-CoV-2 variants and it has emerged as the most prevalent SARS-CoV-2 variant in United Kingdom in end Dec. 2020 (19). At end Dec. 2020, B.1.1.7 variant was found in United States.

### Beta (B.1.351)

B.1.351, commonly known as Beta variant has numerous spike mutations. In October 2020, it was first reported in South Africa, resulting in the 2nd wave of COVID-19 (20). At end Jan. 2021, it was detected in the United States. It has higher transmission risk and decrease neutralization by convalescent/post-vaccination sera and monoclonal antibody treatment (21).

### Gamma (P.1)

P.1 variant, also termed as Gamma variant, was discovered in Brazil in Dec. 2020 and in Jan. 2021, it was reported in United States (22). It may have decrease neutralization by convalescent/post-vaccination sera and monoclonal antibody treatment (21).

### Delta (B.1.617.2)

The fourth variant of concern, B.1.617.2 (also known as the Delta variant), was first found in India in December 2020 and was responsible for the deadly second wave of COVID-19 infections in India in April 2021. This variant was first discovered in the United States in March 2021. Previously, Delta variant was considered a VOI but its rapid global spread triggered WHO to categorize it as VOC in May 2021. This variant spread widely in United States and much of Europe.

### Omicron (B.1.1.529)

From South Africa on 24th Nov. 2021, B.1.1.529 variant was first reported to WHO. On 26^th^ Nov. 2021, WHO declared the variant B.1.1.529 as a variant of concern, termed it Omicron (23). Italian scientists from Bambino Gesu hospital, Rome published the first 3D Omicron image certifying reports that Omicron is highly mutated. It has more mutations than Delta variant. The 3D image revealed that it has several mutations concentrated in spike (S) protein (part which facilitates it in entering human cells (24). Cases of Omicron have been found in Botswana, Israel, Hong Kong. Belgium reported, Europe's first case of Omicron variant. Germany, Italy, UK reported their two cases of Omicron on 27th Nov. 2021. Denmark on 28th Nov. reported two cases of Omicron in travelers from South Africa. It has sparked global concern and widespread travel curbs.

## Variants of Interest (VOIs)

Variants having specific genetic markers have been linked to changes which may result in increased virulence or transmissibility, decreased neutralization of antibodies gained from vaccination or infection, the capability to avoid detection or a reduction in therapeutic or efficacy of vaccines are termed as VOIs. On 22 June 2021, WHO published an epidemiological update that identified 7 VOIs which are mentioned below:

### Epsilon (B.1.427 and B.1.429)

Epsilon (B.1.427 and B.1.429) variants, first appeared in the United States in June 2020 and multiplied from 0 to >50% of reported cases between 1st September 2020 and 29th January 2021, with an 18.6–24% enhancement in transmission as compared to the previous circulating strains. Precise mutations are present in these strains. CDC has designated this variant as a variant of concern in the United States because of its enhanced transmission rate (25).

### Zeta (P.2)

Zeta (P.2) variant was identified in April 2020 in Brazil and exhibits spike mutations. WHO and CDC have designated this variant as VOI because of its possible decrease in neutralization of antibody therapies and vaccines efficacy.

### Eta (B.1.525) and Iota (B.1.526)

Eta (B.1.525) and Iota (B.1.526) variants, were found in New York in Nov. 2020 and have spike mutations. CDC and WHO have categorized these variants as VOIs because of their possible decrease in neutralization of antibody therapies and vaccines efficacy.

### Theta (P.3)

Theta (P.3) variant, also known as GR/1092K.V1, have spike mutations and was first found in Japan and Philippines in Feb. 2021 and is categorized as VOI by WHO.

### Kappa (B.1.617.1)

Kappa (B.1.617.1) variant was discovered in Dec. 2021 in India and is designated as a VOI by CDC and WHO.

### Lambda (C.37)

Lambda (C.37) variant was initially found in Peru. WHO categorized it as variant of interest in June 2021 because of its high prevalence in South America.

## Function of Replication in Pathogenicity

**Figure 2:**
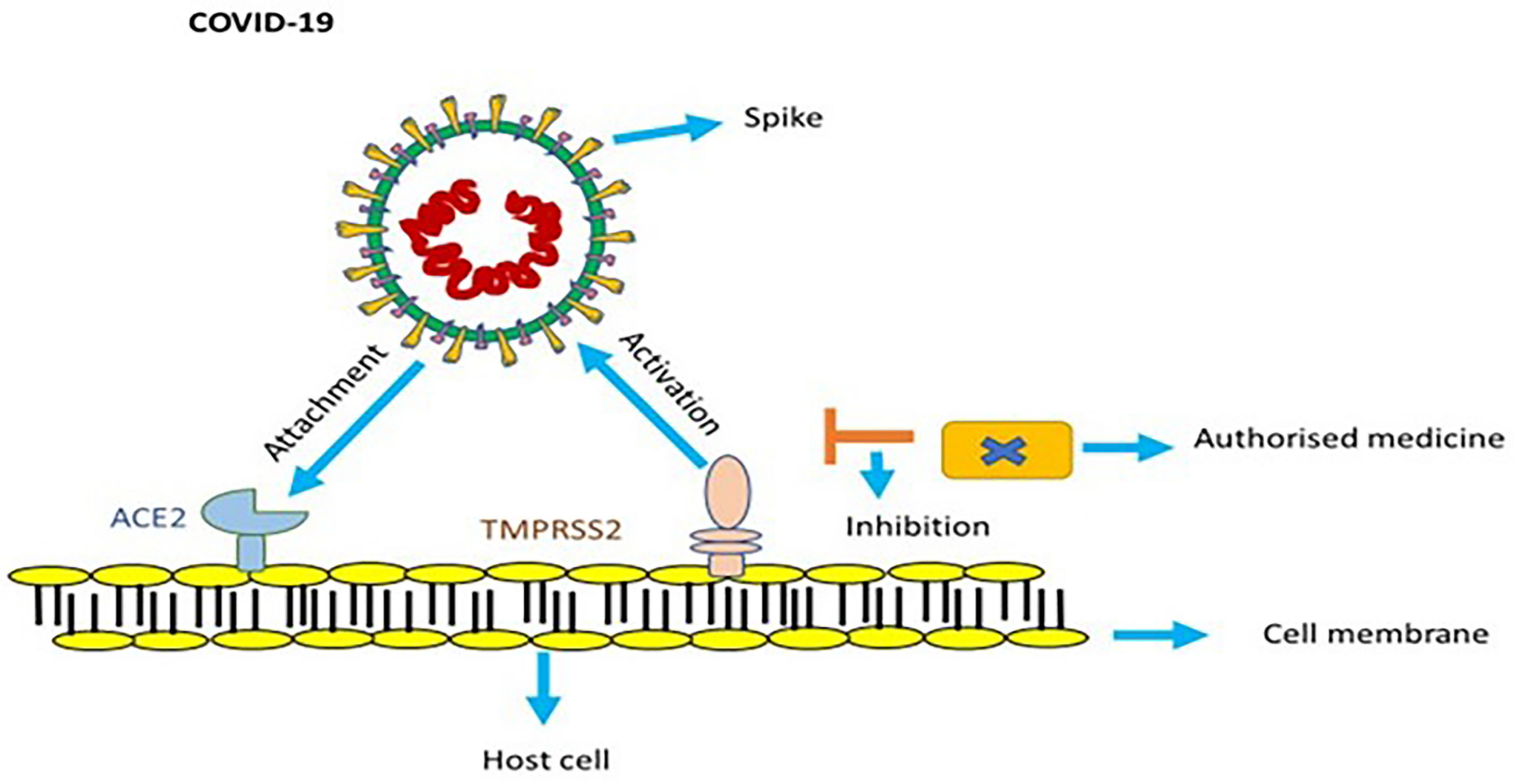
In order to get activated, spike of COVID-19 utilizes ACE2 and TMPRSS2 protease. Endorsed medicines targeted to counter TMPRSS2 prevent infection of COVID-19.

SARS-CoV-2 binds with angiotensin-converting enzyme 2 (ACE2) with the help of its spikes, which help it enter a cell and cause an infection (Figure 2). For it to make a full entry, the glycoprotein spike must be primed by an enzyme protease called TMPRSS2. This protease is essential to the spike's attachment to ACE2 (26, 27). Upon entering the host cell, the viral genome RNA is discharged into the cytoplasm; the genome is first transcribed, and it is later translated. Genomic replication and transcription occur in the cytoplasmic membrane and involve the synthesis of both continuous and discontinuous RNA. This process is facilitated by a viral replicate, a large complex protein encoded with a 20-kb replicase gene (28). It is assumed that a replicase complex comprises numerous cellular proteins and around 16 viral subunits. At the cell membrane, proteins are assembled and as the complete particles are created by budding through internal cell membranes, genomic RNA is assimilated (29).

## Cross Species Transmission

There is a close relation amongst coronaviruses found in domestic animals and human beings and they have transpired in recent times at a climbing rate. Higher animal density habitats that promote interactions between interspecies like farms, markets, shelters and kennels, are related with their emergence, increasing disease incidence and promoting cross-species transmission. Furthermore, animals kept in inferior surroundings or subjected to stress (for example, during transportation) have poor health and repressed immune systems, making them further prone to illnesses (30). In mink fur farms, animals are housed in cramped, unsanitary enclosures, generating novel strains of SARS-CoV-2 triggering secondary zoonoses (31, 32). Wet markets and trading of wild animals also contribute to emergence of diseases as it involves animals transportation and also because they are housed in overcrowded, small and unclean cages near to various other animal species (33). An investigation revealed that in comparison to civets on supply farms, civets in markets were strangely positive for SARS-CoV-1 (34). Hence, the notion of “one health” is vital for containing the coronavirus outbreak.

## Transmission and Universal Epidemiology

Human beings of all ages are prone to infection. Generally, the occurrence of a contagious disease encompasses three important factors: source of infection, route of transmission, and receptive inhabitants (35). The COVID-19 epidemic has a diverse trend of spreading from initial infections (probably from the common source of origin, the Huanan seafood marketplace) to the later cases of human-to-human transmission seen in epidemic curves (36). COVID-19 spreads easily amongst human beings who are in close contact of each other (within 2 meters or 6 feet). There are many means of transmission. The leading cause of transmission of infection in humans principally occurs via droplets produced during sneezing and coughing by symptomatic persons. Nearby persons (within 6 feet) can inhale these droplets or they can land on their mouth, nose and eyes causing infection. It can also spread by very tiny droplets or SARS-CoV-2 aerosols which can remain viable in air having a 1-h half-life causing airborne transmission (37). It can also be spread by asymptomatic persons, even prior to the emergence of any symptoms (20). In both symptomatic and asymptomatic persons, there tend to be larger viral loads in the nasal cavity than in the throat (38). There is also the probability of fecal–oral transmission, as SARS-CoV-2 has been found in fecal samples and anal swabs (39). A few persons have been found to be “super spreaders” (i.e., they have infected more than 10 persons) of this virus (2). The fact that 3,019 health care persons became infected as of February 12, 2020 points also to nosocomial transmission routes (40) and there have been reports of neonatal illness from transmission during the postnatal phase (41). Additionally, it is noticed in tears of coronavirus patients having conjunctivitis, implying that transmission may also occur by way of ocular infection. In all cases, patients can be contagious both before the emergence of symptoms and in postrecovery (42).

In susceptible populations, the basic reproductive number speaks to the number of infections generated by each infected person. This number (R_0_) for SARS-CoV-2 is in the range of 2.2–2.6, that of SARS-CoV ranges from 1.4 to 5.5, and that of MERS-CoV exceeds 1 (43). The basic case reproduction rate (BCR) of the H1N1 flu is 1.3, while that of SARS is 2. The BCR of SARS-CoV-2 is higher, signifying that it has greater chances of transmission and can thus more easily create a pandemic. In different modeling investigations, the BCR of SARS-CoV-2 is projected to vary from 2 to 6.47 (44). Table 2 compares the epidemiological attributes of SARS-CoV, MERS-CoV, and SARS-CoV-2.

**Table 2:** An analogy of epidemiological features of SARS-CoV, MERS-CoV and SARS-CoV-2.

**Outbreaks**	**SARS-CoV**	**MERS-CoV**	**SARS-CoV-2**
Assessed R_0_ (Reproductive No.)	1.4–5.5	>1	2.2– 2.6
Human to human transmission	Nosocomial, aerosols, zoonotic, fecal-oral transmission	Aerosols, zoonotic, respiratory, nosocomial, limited human-to-human transmission	Close contact, nosocomial, zoonotic, aerosols transmission
Incubation phase (Days)	4.6	5.2	6.4
Primary host	Chinese horseshoe bats	Bats	Bats
Secondary host	Masked palm civets	Camels	Pangolins
Tertiary host	Human beings	Human beings	Human beings

**Figure 3:**
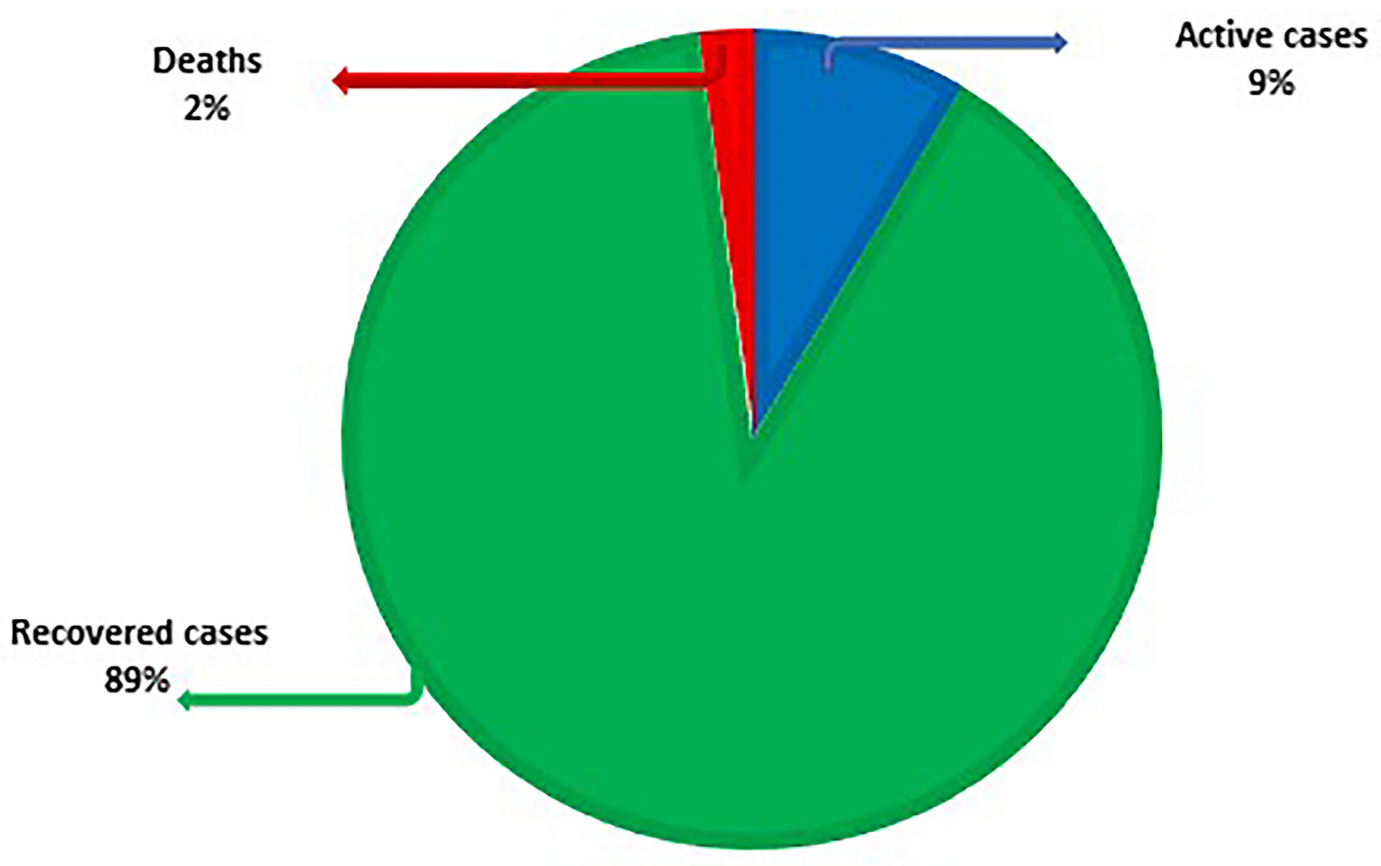
Allocation prototype for recovered, active cases and deaths globally as on 2nd Sep 2021.

As of the time of writing, COVID-19 has reached 221 nations. As on 2nd Sept, 2021, there were 219,923,515 lab-confirmed cases, 196,562,686 recovered cases (89%), 18,805,002 active cases (9%) and 4,555,827 deaths (2%) (Figure 3). Table 3 lists the eight countries with the highest number of lab-confirmed COVID-19 cases as on 2nd Sept, 2021 (45).

**Table 3:** Allocation of Lab confirmed cases of eight most affected countries worldwide by COVID-19 and their death rate as on 2nd Sept 2021.

**No**.	**Countries**	**Reported cases**	**Deaths**	**Recovered**	**Active**	**Death rate**
1	USA	40,513,018	662,853	31,199,835	8,650,330	1.63
2	India	32,902,345	439,916	32,056,085	406,344	1.33
3	Brazil	20,830,712	582,004	19,801,725	446,983	2.79
4	Russia	6,956,318	184,812	6,218,048	553,458	2.65
5	UK	6,862,904	132,920	5,533,227	1,196,757	1.93
6	France	6,799,240	114,680	6,310,859	373,701	1.68
7	Turkey	6,435,773	57,283	5,872,385	506,105	0.89
8	Argentina	5,195,601	112,195	4,884,418	198,988	2.15

## COVID-19 Death Rate by Age

**Figure 4:**
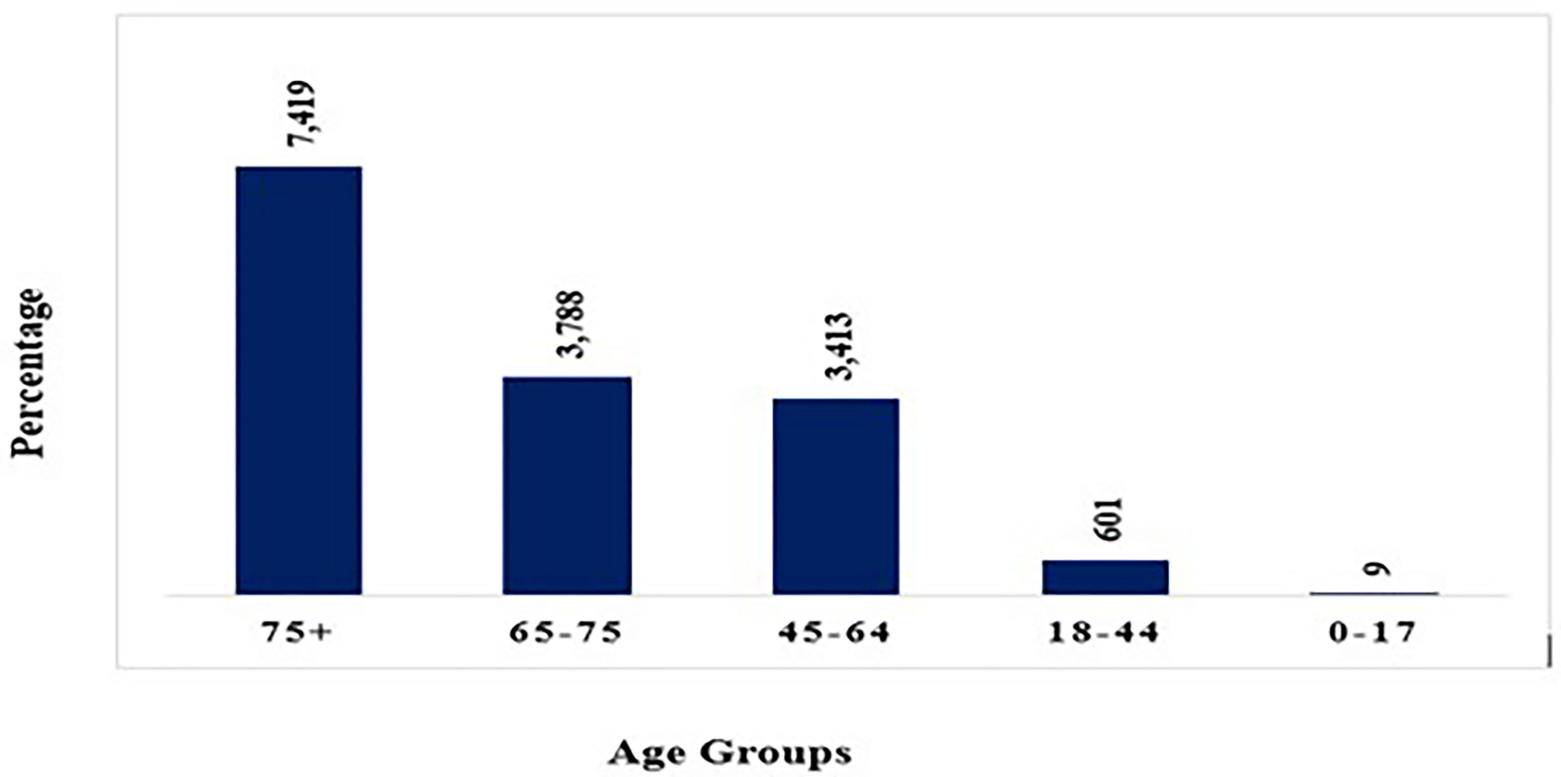
COVID-19 death rate by different age groups.

Various studies reveal that death rate rises as people age. Children below age nine appear to be largely unaffected having none or minor indications whereas persons over the age of eighty and those suffering from chronic illnesses are highly vulnerable. After crossing 80 years of age, around 14.80 percent of affected people died. For people over the age of 50, the fatality rate begins to rise (Figure 4). Infected people below 50 years of age have a 0.4 percent fatality rate whereas those aged 50 to 59 have a 1.3 percent death rate. It is 3.6 percent for those aged 60 to 69, 8 percent for those aged 70 to 79, and 14.8 percent for above 80 years (46, 47).

## COVID-19 Death Rate by Sex

**Figure 5:**
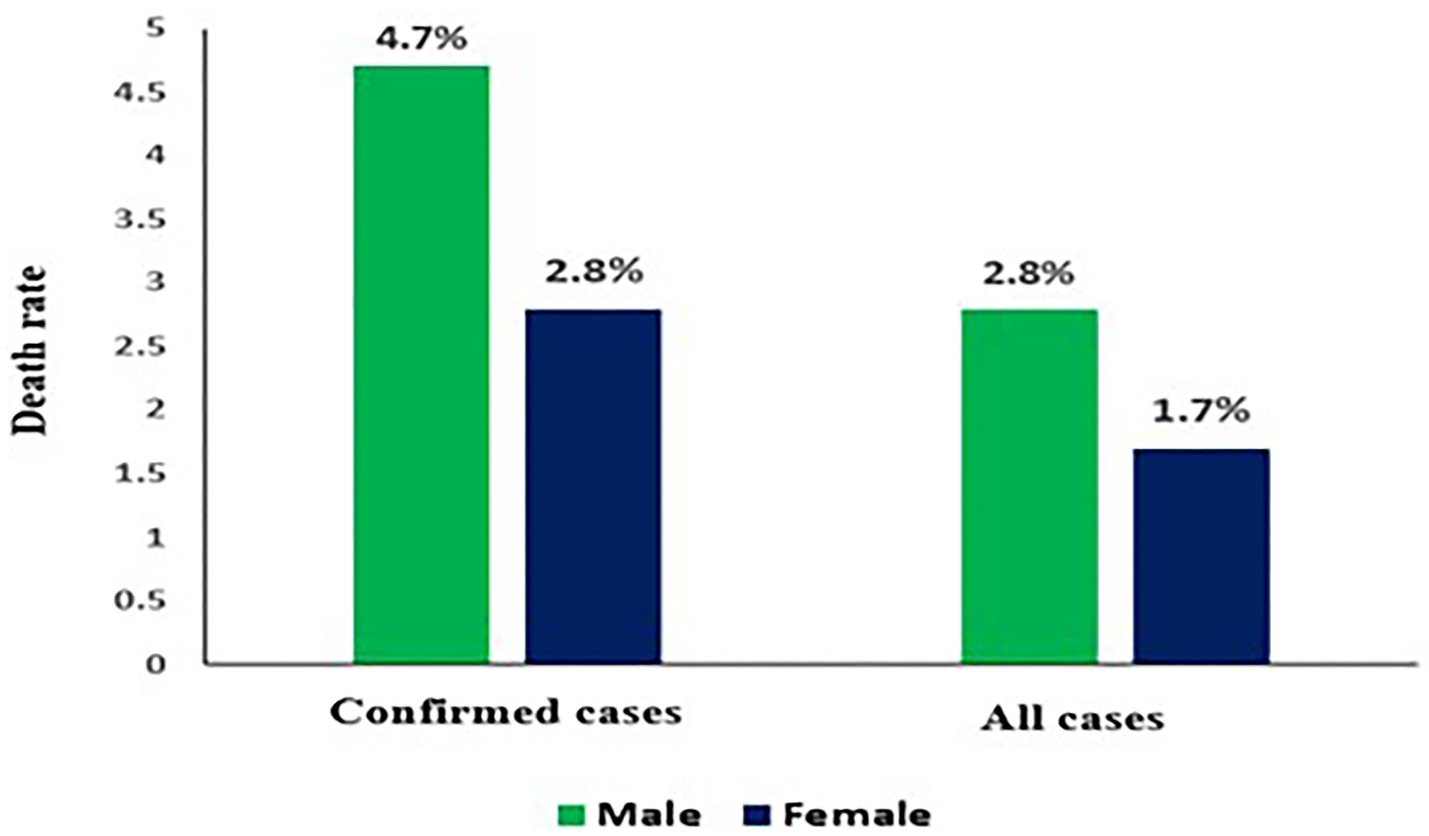
COVID-19 death rate by sex ratio.

As the global fatality rate from COVID-19 rises, it is evident that percentage of men getting extremely ill or dying from COVID-19 is greater as compared to women. However, there is a variation in figures based on country but it did not certainly indicate any biological difference. Researchers are still unaware but since on an average, men are more involved in health damaging behaviors like smoking and drinking than women. Difference in death rates based on sex as shown in (Figure 5) (47).

## Vaccination Status

During 4th April to 17th July, by means of vaccination status, within 13 U.S. jurisdictions, an analysis was done for COVID-19 cases, hospitalizations and fatalities for people aged ≥18 years. The findings state that 92 percent of COVID-19 cases (569,142), 92 percent hospitalizations (34,972) and 91 percent fatalities (6,132) were confirmed in people who were not fully vaccinated and 8 percent cases (46,312), 8 percent hospitalizations (2,976) and 9 percent fatalities (616) were confirmed in fully vaccinated people (48). These findings confirm the immense benefits of full vaccination against COVID-19.

## Diagnosis

A suspect case is any person with cough, sore throat or fever who has visited any area with continuing community or local transmission, or who has come in close contact with any person diagnosed with COVID-19. A suspect case with a positive molecular test is considered a confirmed case. Some persons can be asymptomatic and not have a fever. Precise diagnosis is typically made by undertaking detailed molecular tests on various respiratory samples (e.g., nasopharyngeal/throat swab, bronchoalveolar lavage, sputum, and endotracheal aspirates), although it is sometimes diagnosed through blood and stool sample analysis. Most coronaviruses cause minor upper-respiratory tract illnesses and are limited in nature, and there is typically no need for them to be diagnosed. In the case of COVID-19, however, it is essential that diagnosis be immediate and that infected persons be isolated. A diagnostic workflow for COVID-19 was produced only a few days after reports of the initial cases, and on March 19, 2020, the WHO published provisional directions for lab testing (49, 50). Since then, various lab procedures have been used to detect COVID-19, and details of the related processes have been likewise published (4, 13).

In sites with limited resources, persons should be tested only if confirmed test results demand necessary action. Under other conditions, tests by which to diagnose COVID-19 should be undertaken immediately, particularly among symptomatic medical and health care workers and also in pediatric centers and nursing homes to detect any outbreak in its very early stages. Persons diagnosed through lab-confirmed COVID-19 testing also test positive in real-time reverse transcriptase polymerase chain reaction (RT-PCR) analysis of nasopharyngeal swab, blood, sputum, urine, and stool samples (1).

## Tests for Diagnosing COVID-19

### Real-Time RT-PCR

Since there is no standard reference for identifying SARS-CoV-2, NAATs (nucleic acid amplification tests) like real-time RT-PCR are prime techniques for diagnostic testing of COVID-19 infection (51, 52). Infections are diagnosed by nucleic acid-based polymerase chain reaction (PCR), which magnifies a precise genetic series in the coronavirus as radiological results, and so symptoms are not specific. Molecular tests based on RT-PCR denotes gold standard procedures globally for making a confirm COVID-19 infection diagnosis (53). These molecular tests are ideal diagnostic choice for broad surveillance policies as there are low costs for the extraction of complete viral RNA, reverse transcription, amplification process and accessibility of thermal cyclers for RT-PCR in labs, hospitals, and research centers (54).

### Rapid Antigen/Antibody Tests

As the pandemic grew, it became essential to implement rapid and economical diagnostic testing methods to perform vast surveillance operations (55). In order to deal with it, rapid antigen/antibody tests were developed to identify viral antigens or anti-SARS-CoV-2 antibodies in blood samples and nasal as well as salivary swabs. These tests are performed for regular testing of hospital or school staff as they are working in at-risk conditions or for vast screening of residents in case there are chances of a new disease outbreak (56). These tests are prompt and carried out in 15 to 30 min, easier to perform and economical in comparison to RT-PCR (57). The tests are developed on the model of LIFA (lateral flow immunoassay) for detecting viral proteins in case of rapid antigen tests or human antibodies contrary to SARS-CoV-2 antigens in case of rapid antibody tests.

### Immunoenzymatic Serological Tests

Immunoenzymatic serological tests used for detecting COVID-19 are developed based on indirect ELISA (enzyme-linked immunosorbent assay). It is a chemiluminescent assay used for identification and quantitation of immunoglobulins, human proteins, peptides, and antigens by means of binding amongst a particular antibody and its target protein which produces a noticeable indication. The prime benefit of this technique is that it generates sensitive and precise results in 1 to 5 h (58). ELISAs are presently used for detecting IgG and IgM antibodies (59) particularly for SARS-CoV-2 antigens or for detecting spike proteins of virus (60). Recently, few ELISAs have been created for detecting human IgA antibodies whose detection is of prime significance since these are the first antibodies generated after COVID-19 infection (59). Specifically, ELISA tests for identification of IgM and IgG antibodies are administered on patients who got a negative outcome in molecular testing done on nasopharyngeal swabs to determine the patient's seroconversion and gaining immunocompetence contrary to COVID-19 infection (61). ELISAs for identifying viral proteins and human IgA antibodies are used for wide screening policies and diagnostic testing since these molecules are instantly detected in clinical samples (62).

## Prevention and Treatment

Prevention is crucial to containment. COVID-19 has a number of characteristics that make prevention difficult (e.g., transmission from asymptomatic people, longer incubation time, infection before the emergence of symptoms, longer disease period, and the possibility of transmission after recovery). Various previous outbreaks like those involving Nipah (63), Zika (64) and SARS taught us that avoiding contact with infected people, regular hand washing with soap and water, maintaining sufficient distance from other people, wearing protective face masks, and refraining from touching one's nose and mouth, especially when outside, are the best ways to protect oneself. Hand sanitizers can also be used as an alternative to handwashing. Like other respiratory diseases, COVID-19 can be transmitted from infected persons to others via droplets, infected body parts, and infected objects. As per WHO recommendations, close contact should be avoided with patients or farm or wild animals (41). Persons who have become infected can amplify it and transmit it to others, and so to mitigate viral proliferation, they should take all necessary precautions (e.g., cough hygiene, maintaining sufficient distance from others, regular handwashing with soap and water, isolating themselves from other family members, and safely disposing of contaminated objects). Immunocompromised persons in particular should avoid public congregations, as it has been found that containment of this virus is best achieved through self-isolation. Those who cannot, including hospital, emergency medicine, and diagnostic labs personnel should follow strict hygiene practices and approved infection prevention and regulatory procedures. Furthermore, health care workers should use N95 masks, gloves, eye protection, personal protective equipment kits, and medical gowns. Recent treatments are based on prior practices pertaining to the treatment of SARS, MERS, Ebola, influenza, and other viral respiratory diseases. Hence, treatment of COVID-19 is generally supportive and symptoms-based, and frequently features oxygen therapy.

First, prior to treatment, any confirmed case should be completely isolated, in order to stop the infection from spreading to other persons. Patients with mild symptoms can be treated at home, as per guidelines issued by the health departments of various countries; in such cases, managing fever and cough and having adequate hydration and nutrition are essential. General antivirals and antibiotics must be directed to COVID-19 patients. Noninvasive ventilation, oxygen in high-flow nasal canula, and nasal prongs should be administered to hypoxic patients. Critically ill patients may require extracorporeal membrane oxygenation glucocorticoid therapy, mechanical ventilation, and convalescent plasma therapy (40). Remdesivir, an antiviral that has been effective in prophylaxis and remedial treatment, has also been used (9). Finally, to prevent COVID-19 at the individual level, various kinds of vaccines are available.

## Vaccines Approved by WHO

The vaccines approved by WHO are Pfizer/BioNTech BNT162b2 which got approval in 107 countries, Moderna mRNA-1273 which got approval in 77 countries, Janssen (Johnson & Johnson) Ad26.COV2.S which got approval in 78 countries, Oxford/AstraZeneca AZD1222 which got approval in 125 countries, Covishield (Oxford/AstraZeneca formulation) which got approval in 46 countries, Bharat Biotech (Covaxin) which got approval in 10 countries, Sinopharm (Beijing) BBIBP-CorV (Vero Cells), which got approval in 68 countries, Sinovac (CoronaVac) which got approval in 43 countries (65). Table 4 lists the major COVID-19 candidate vaccine platforms in clinical evaluation (66).

**Table 4:** Major COVID-19 candidate vaccine platforms in clinical evaluation (Produced with permission (66).

**Vaccine name**	**Vaccine platform**	**Developer**	**Clinical trial phase**	**Clinical trial registrations**
BNT162b1/BNT162b2	RNA-based vaccine	Pfizer-BioNTech, Fosun Pharma	Phases I–III in USA, Germany, and China	NCT04368728, NCT04380701, NCT04523571
mRNA-1273	RNA-based vaccine	Moderna, NIAID	Phases I–III in USA	NCT04470427, NCT04405076, NCT04283461
INO-4800	DNA plasmid vaccine	Inovio Pharmaceuticals, International Vaccine Institute	Phases I–III in USA	NCT04447781, NCT04336410
GX-19	DNA plasmid vaccine	Genexine Consortium	Phases I and II in South Korea	NCT04445389
ChAdOx1 nCov-19 (AZD1222)	Adenovirus vector, non-replicating	University of Oxford, AstraZeneca	Phases I–III in UK, South Africa, USA and Brazil	NCT04324606, ISRCTN89951424, EudraCT2020-001228-32, PACTR202006922165132, EudraCT2020-001072-15
Ad26.CoV2-S	Adenovirus vector, non-replicating	Johnson & Johnson	Phases I–III in USA and Belgium	NCT04436276 NCT04505722 NCT04535453 NCT04509947
Ad5-nCoV	Adenovirus vector, non-replicating	CanSino Biologics Inc., Beijing Institute of Biotechnology	Phases I and II; phase II studies in China and Canada	ChiCTR2000031781, ChiCTR2000030906, NCT04341389 NCT04313127
Gam-COVID-Vac	Adenovirus vector, non-replicating	Health Ministry of the Russian Federation	Phases I–III in Russia	NCT04530396 NCT04436471 NCT04437875
PiCoVacc	Inactivated SARS-CoV-2	Sinovac Biotech	Phases I–III; phase III in China and Brazil	NCT04456595, NCT04383574, NCT04352608
COVID-19 vaccine	Inactivated SARS-CoV-2	Sinopharm, Wuhan Institute of Biological Products Co. Ltd	Phases I–III in China	ChiCTR2000034780, ChiCTR2000031809
BBIBP-CorV	Inactivated SARS-CoV-2	Sinopharm, Beijing Institute of Biological Products Co. Ltd	Phases I–III in China and United Arab Emirates	ChiCTR2000034780, ChiCTR2000032459
SCB-2019	Protein subunit	Clover Pharmaceuticals,GlaxoSmithKline, Dynavax	Phase I in Australia	NCT04405908
NVX-CoV2373	Protein subunit	Novavax	Phases I–III in Australia, USA and UK	NCT04368988 NCT04583995 NCT04533399

## Long-Term Consequences of COVID-19

**Figure 6:**
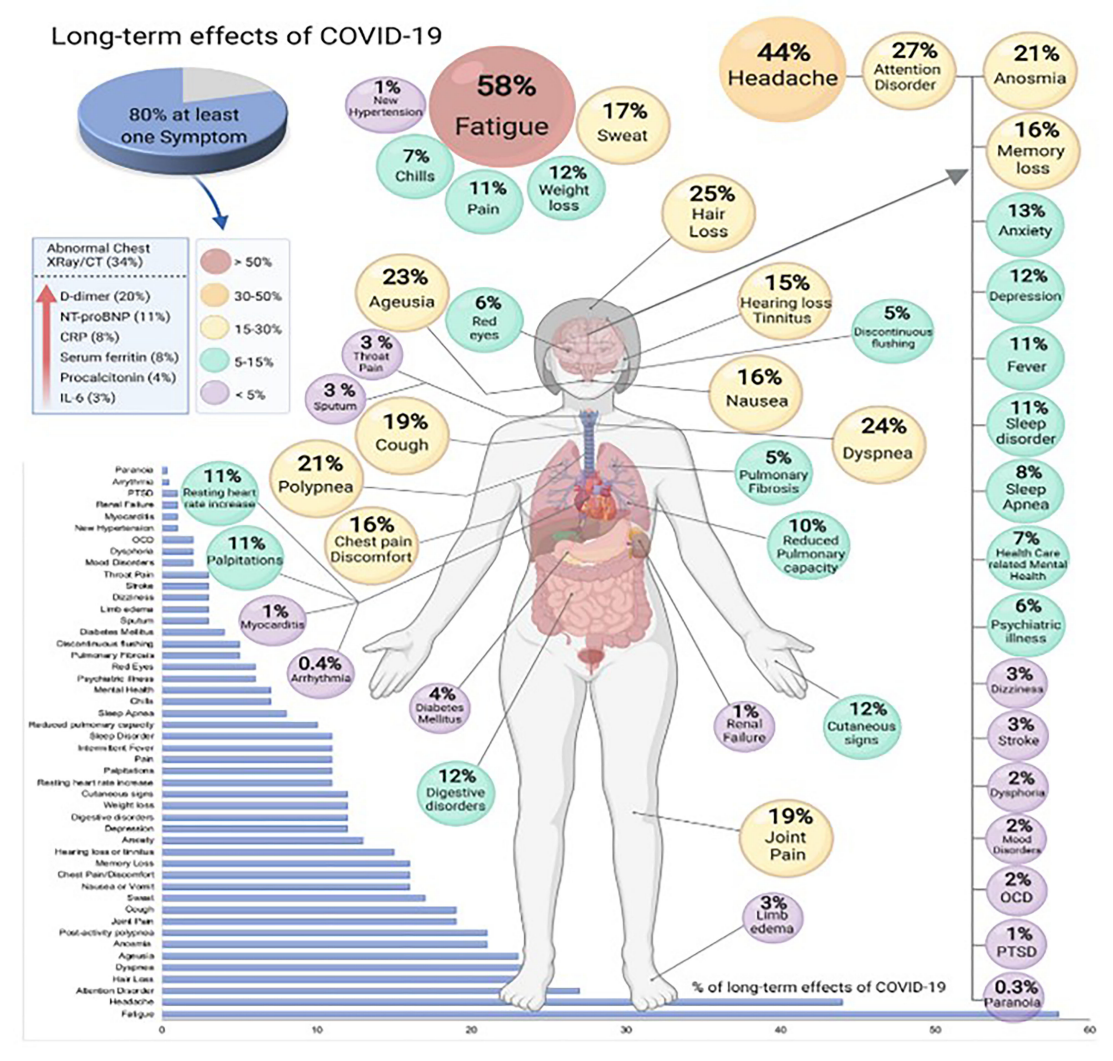
Long-term effects of COVID-19 (produced with permission) (71).

Patients continue to suffer, weeks as well as months following acute Covid 19. With a mortality rate of <0.2 percent, focus is shifted to COVID-19 patients who suffer lasting symptoms for >3–4 weeks (Long Covid). As per a latest study, one in five patients has continual symptoms following 5 weeks and 1 in 10 has for more than 12 weeks or even longer. According to a study done in Italy, post COVID-19 recovery, out of 143 patients, 1, 2, 3, or more symptoms kept on persisting in 32 percent and 55 percent of patients respectively while 13 percent do not have any symptoms (67). Notably, life quality worsened as observed by 40 percent of patients (68). Patients who have superb health and a decent life quality before Long Covid express despair as their symptoms worsens. Patients have described a wide range of lasting symptoms, consisting of difficult breathing, chronic cough, tightness in chest, cognitive impairment, and excessive fatigue. A few patients report about their cyclic condition, with some symptoms getting better while others get worse (69). Most symptoms are neurological like brain fog, unable to concentrate, unable to recall names, head pounding. Some general neurological symptoms which usually occur along with breathlessness and fatigue are cognitive dulling, dizziness, and headaches (69). Psychological symptoms like anxiety, depression, post-traumatic stress, and cognitive decline could be due to neurobiological damage. Symptoms like ageusia, dizziness, anosmia, seizures, and headaches may last long following acute COVID-19 (70). A meta-analysis study showed that 80 % of patients have at least one lasting symptom following 2 weeks of acute COVID-19. In sum, 55 effects consisting of signs, symptoms and lab constraints were detected with anosmia, fatigue, irregular chest X-ray/CT, lung disfunction and neurological ailments were the common most (Figure 6) (71).

## Global Impact

The current pandemic corresponds to the greatest threat to human lives, their livelihoods, and global economies after World War II. The pandemic has sent the globalization processes into reversal. The social and economic disorder triggered by this pandemic is destructive. As per WHO, tens of millions of people run the risk of plunging into severe poverty. It has destroyed jobs, putting millions at risk of losing their livings. There is a sharp increase in unemployment rates globally (72). New job openings are very low. As per an IMF (International Monetary Fund) estimation, world economy has shrunk by 4.4 percent in 2020. IMF has mentioned it as the worst fall ever since the Great Depression of 1930. All the global share markets have witnessed huge falls as COVID-19 cases grew in the early months of pandemic and most of the countries went into recession.

The aviation industry has been hit hard with airlines making flight cuts. Data from Flight radar 24 indicates that, No. of daily flights were hit hard in 2020 globally and is still far from recovery. Millions of jobs in hospitality sector were lost. All the leading travel destinations have registered a huge fall in hotel reservations. The tourism sector has collapsed globally (73). In 2020 alone, billions of dollars were lost and as per industry analysts, global tourism would not be able to return to pre pandemic levels till 2025. There is a huge drop in retail footfall as customers preferred to stay at home. Due to a change in shopping behavior, there is an unprecedented surge in online shopping having a worldwide revenue of 3.9 trillion dollars in 2020. Billions of dollars have been pledged for COVID-19 vaccine and treatment by governments of many countries. Shares of few pharmaceutical companies developing vaccines have increased. Several companies have started distributing vaccines and many countries have started vaccination programs (73, 74). 54.4 percent of the global population has received a minimum of 1 dose of COVID-19 vaccine with 7.98 billion vaccine doses been administered worldwide (75).

## Strategies Implemented for Controlling COVID-19 Pandemic

For controlling COVID-19 pandemic; screening, suppression/containment as well as mitigation are the strategies implemented. Screening is performed using a thermometer for detecting a higher body temperature related with fevers caused by COVID-19 (76). Containment/suppression was implemented in the initial stages of COVID-19 pandemic with the goal of tracing and isolating persons who have been infected as well as additional measures were implemented to prevent the disease's spread. When containing the pandemic became no longer viable, efforts were shifted to mitigation, which involves taking steps to reduce the disease's spread and mitigate its impact on the society and medical facilities. For effective mitigation (i) transmission chains were destroyed swiftly by means of screening as well as containment, (ii) medical facilities were provided to infected persons on an immediate basis (c) contingencies were made allowing for the efficient implementation of (i) and (ii). At same time, a combination of containment as well as mitigation strategies were implemented (77). Suppression which necessitates more drastic measures was implemented for decreasing the basic reproduction number to less than one, to stop the pandemic from spreading (78).

## Future Directions to Prevent COVID-19

The subsequent forward-looking aspects should be taken into consideration. To begin with, it's possible that, like mRNA vaccines, other vaccines are as well helpful in providing protection from COVID-19 but not from infection and in this case, vaccines can get infected and can circulate the virus easily. Wearing protective face masks and maintaining sufficient distance from other people will be a prime requirement to contain this pandemic. Secondly, how long will the vaccine induced immunity last? Do we require booster dosages periodically? This question is also related to patients who have recovered from COVID-19. It is necessary to follow up the vaccines to solve this issue.

Thirdly, it is (SARS-CoV-2) is an RNA virus, which can mutate easily to avoid immunological pressure. In terms of immune selection, current evidence suggests that Delta and Omicron variant will pose a significant challenge in the creation of a vaccine that will provide universal protection from all variants of SARS-CoV-2. Even if new vaccines are developed, to deal with Delta and Omicron variants, there is a strong possibility that other dangerous variants will keep on evolving and will emerge with further immunological pressure. In the coming years, in this direction, we can look forward to a range of new data. A recent report mentioned, intense selection (SARS-CoV-2) in convalescent plasma treatment, which was linked to the generation of viral variants with evidence of decreased vulnerability with respect to neutralizing antibodies (79). Understanding the interaction amongst the humoral immune response and viral adaptability will be vital in developing immunotherapy and an improved vaccine having universal coverage for COVID-19 (66).

## Conclusion

Over the last 50 years, various coronavirus types that instigate numerous human and animal diseases have emerged. There is strong likelihood that such coronaviruses will continue to emerge, and to evolve and trigger both human and animal outbreaks; this likelihood is driven by these viruses ability to mutate, recombine, and cause infection in human beings and animals alike. Future research will continue to examine various facets of replication among these coronaviruses, and their pathogenesis and mutation. To understand where and when an epidemic will likely emerge, we need to understand the tendency of these coronaviruses to transmit amongst various species and their ability to infect new hosts; we also need to determine their most important reservoirs. It is likely that bats are important reservoirs of these viruses, and so it is important to establish how humans can block viral transfers to other animal species and clinically evade these diseases. Moreover, various nonstructural and additional proteins encoded by these viruses remain unspecified and have as-yet undefined purposes, and so it is of paramount importance that scientists detect the processes in which these unidentified proteins are involved and determine their contributions to the pathogenesis and replication of these viruses. Such research should aim to increase the number of appropriate therapeutic means of fighting the infections they cause. In conclusion, fully elucidating the complete processes by which COVID-19 causes disease and determining the nature of host immunopathological responses will substantially advance our ability to find effective treatments for this disease. These will be made possible through the undertaking of clinical trials and the creation of approved medicines that combat this virus.

## Author Contributions

All authors listed have made a substantial, direct, and intellectual contribution to the work and approved it for publication.

## Funding

The authors extend their appreciation to the Deanship of Scientific Research at King Khalid University, Abha, Saudi Arabia for funding this work through research groups program under grant number RGP.1/145/42.

## Conflict of Interest

The authors declare that the research was conducted in the absence of any commercial or financial relationships that could be construed as a potential conflict of interest.

## Publisher's Note

All claims expressed in this article are solely those of the authors and do not necessarily represent those of their affiliated organizations, or those of the publisher, the editors and the reviewers. Any product that may be evaluated in this article, or claim that may be made by its manufacturer, is not guaranteed or endorsed by the publisher.
